# Low molecular weight sulfated chitosan efficiently reduces infection capacity of porcine circovirus type 2 (PCV2) in PK15 cells

**DOI:** 10.1186/s12985-022-01781-7

**Published:** 2022-03-24

**Authors:** Daniela Jiménez-Arriagada, Alejandro A. Hidalgo, Victor Neira, Andrónico Neira-Carrillo, Sergio A. Bucarey

**Affiliations:** 1 Programa de Doctorado en Ciencias Silvoagropecuarias y Veterinarias, Campus Sur Universidad de Chile, Santa Rosa 11315, La Pintana, CP: 8820808 Santiago, Chile; 2 Escuela de Química y Farmacia, Facultad de Medicina, Universidad Andres Bello, Sazié 2320, Santiago, Chile; 3 Unidad de Virología, Departamento de Medicina Preventiva, Facultad de Ciencias Veterinarias y Pecuarias, Universidad de Chile, Santa Rosa 11735, La Pintana, Santiago, Chile; 4 Laboratorio Polyform, Departamento de Ciencias Biológicas, Facultad de Ciencias Veterinarias y Pecuarias, Universidad de Chile, Av. Sta. Rosa 11735, La Pintana, Santiago, Chile; 5 Departamento de Ciencias Biológicas, Centro Biotecnológico Veterinario, Biovetec, Facultad de Ciencias Veterinarias y Pecuarias, Universidad de Chile, Santa Rosa 11735, La Pintana, Santiago, Chile

**Keywords:** Chitosan, Antiviral polymers, Sulfated chitosan, Porcine circovirus type 2, PK15 cells, Cell attachment

## Abstract

**Background:**

Porcine circovirus type 2 (PCV2)-associated diseases are a major problem for the swine industry worldwide. In addition to vaccines, the availability of antiviral polymers provides an efficient and safe option for reducing the impact of these diseases. By virtue of their molecular weight and repetitious structure, polymers possess properties not found in small-molecule drugs. In this perspective, we focus on chitosan, a ubiquitous biopolymer, that adjusts the molecular weight and sulfated-mediated functionality can act as an efficient antiviral polymer by mimicking PCV2-cell receptor interactions.

**Methods:**

Sulfated chitosan (Chi-S) polymers of two molecular weights were synthesized and characterized by FTIR, SEM–EDS and elemental analysis. The Chi-S solutions were tested against PCV2 infection in PK15 cells in vitro and antiviral activity was evaluated by measuring the PCV2 DNA copy number, TCID50 and capsid protein expression, upon application of different molecular weights, sulfate functionalization, and concentrations of polymer. In addition, to explore the mode of action of the Chi-S against PCV2 infection, experiments were designed to elucidate whether the antiviral activity of the Chi-S would be influenced by when it was added to the cells, relative to the time and stage of viral infection.

**Results:**

Chi-S significantly reduced genomic copies, TCID50 titers and capsid protein of PCV2, showing specific antiviral effects depending on its molecular weight, concentration, and chemical functionalization. Assays designed to explore the mode of action of the low molecular weight Chi-S revealed that it exerted antiviral activity through impeding viral attachment and penetration into cells.

**Conclusions:**

These findings help better understanding the interactions of PCV2 and porcine cells and reinforce the idea that sulfated polymers, such as Chi-S, represent a promising candidates for use in antiviral therapies against PCV2-associated diseases. Further studies in swine are warranted.

## Background

The chitosan (Chi) biopolymer is a derivative obtained from chitin, which is a natural polysaccharide, poly-β-(1,4)-d-glucosamine-N-acetyl-d-glucosamine, present in the cell walls of fungi, yeasts, and in the exoskeleton of invertebrates [1]. The main source of chitin corresponds to crustacean shells and insect cuticles [2]. It is possible to obtain Chi from partially deacetylated chitin (50%), which corresponds to a linear cationic biopolymer formed by glucosamine and N-acetyl d-glucosamine subunits, which are chemically linked by covalent O-glycosidic β-(1–4) bonds [3, 4]. The molecular weight of Chi ranges between 50 and 1000 kDa with a degree of deacetylation > 50% [2, 5, 6].

Chemical modification of Chi occurs selectively in nucleophilic amino groups (position C-2 in repeating units of glucosamine), or in hydroxyl groups (positions C-3, C-6 in repeating units of acetyl glucosamine and glucosamine) or indistinctly in amino and hydroxyl groups [7]. The chemical surface of Chi can be modified using sulfate groups and their derivatives can produce polyampholitic chains similar to those found in the structure of sulfated glycosaminoglycans (GAGs), which represent a special class of charged polysaccharides involved in the formation of extracellular matrix, such as chondroitin sulfate and heparan sulfate [8]. GAGs can interact with various proteins in the extracellular matrix, thus signaling to mediate various cellular processes, such as adhesion, migration, proliferation and differentiation [9, 10]. GAG sulfates have numerous important pharmacological properties and biological activities such as antiviral and anticoagulant among other effects [11]. Therefore, sulfated GAG derivatives, such as sulfated chitosan (Chi-S), represent prominent candidates for use in several biomedical applications [12–15]. With specific regard to antiviral effect, the material’s degree of sulfation (DS) (i.e., number of sulfate groups per monosaccharide unit) is an important parameter for antiviral activity [16]. There is a positive correlation between increasing DS and antiviral potency [17]. In the cases of several human viruses, including HIV, it was thought that sulfated polysaccharides work through dual mechanisms: blocking viral adsorption and inhibiting viral replication [18]. It is now believed that the predominant mechanism is the inhibition of viral adsorption and syncytium formation [19].

Porcine circovirus type 2 (PCV2) is a small non-enveloped virus, belonging to the genus Circovirus, family *Circoviridae* [20]. PCV2 is the primary causative agent of postweaning multisystemic wasting syndrome (PMWS) and other diseases in swine referred to as porcine circovirus associated diseases (PCVAD) [21]. PMWS was first described in Canada in 1991, and nowadays it is described in pig populations worldwide, producing a serious economic impact on the swine industry. Therefore the development of antiviral options is critical [22].

This work presents the synthesis and characterization of Chi-S and its antiviral effect against PCV2 on PK15 cells (a porcine kidney-derived cell line highly permissive to PCV2 infection), and discuss how these effects depend on its sulfate functionalization, molecular weight and concentration. In addition, to explore the mode of action of the Chi-S against PCV2 infection, experiments were designed to clarify whether the antiviral activity of the low molecular weight Chi-S would be influenced by when it was added to the cells, relative to the time and the stage of viral infection.

## Materials and methods

### Material and equipment

Reagents of the highest available grade were used. All experiments and solution preparations were performed using fresh Milli-Q water (LabostarTM TWF, Evoqua Water Technologies LLC, Warrendale, PA, USA) and Ultra-pure water (18.2 MΩ) was obtained from the LaboStar™ 4-DI/UV water system. Fourier transform infrared spectra analysis (FT-IR) was performed on a ATR/FT-IR interspec 200-X spectrometer (Interspectrum OU, Toravere, Estonia). Scanning electron microscopy—energy dispersive X-ray spectroscopy (SEM–EDS) analysis of Chi-S and Chi-C samples was performed in a JEOL JSM-IT300LV microscope (JEOL USA Inc., Peabody, MA, USA), connected to an energy dispersive X-ray detector for elemental analysis with computer-controlled Aztec EDS system software from Oxford Instruments, Abingdon, UK. Real-time PCR reactions were performed using the LightCycler® Nano Instrument (Roche Molecular Systems, Inc). High resolution for PCV2 viral copy number determination and melting curve analysis were achieved with this instrument.

### Synthesis of sulfated chitosan

To obtain Chi-S, commercial Chi (Sigma-Aldrich) of two different molecular weights was used. Low molecular weight (LMW) Chi (50–190 kDa) and high molecular weight (HMW) Chi 310–375 kDa were subjected to a sulfation reaction using the modified method described by Naggi et al. [23]. Briefly, 5 g of each Chi was dissolved in 200 mL of cold sulfuric acid (Sigma-Aldrich) (4 °C) and stirred for 2 h. Then the mixture was precipitated dropwise in 1 L of cold ethyl ether (Sigma-Aldrich) (2 °C) under constant stirring. The precipitated product was filtered, washed with abundant cold ethyl ether, and products were collected by adding them to 200 mL of ultra-pure chilled water (4 °C). The water was obtained from first capsule filters with 0.2 µm flow (U.S. Filter), and ultra-pure water (18.2 M Ω) was obtained from the LaboStar TM 4-DI/UV water system. The pH was adjusted to 7.6 with a cool 30% NaOH aqueous solution (Merck) and the mixture was dialyzed against distilled water on Spectra/Por 3 cellulose membranes with 3.5 kDa pore size for 3 days at room temperature. Distilled water was obtained from an automatic water still, model 700-Pobel. The volume of dialysis solution was changed every 24 h. Finally, the product obtained was subjected to evaporation under reduced pressure in a rotary evaporator (Heidolph Laborotta 4001 Efficient), until obtaining 20 mL, which was lyophilized using the Christ Alpha 1–4 equipment, LOC-1M.

### Characterization of chitosans

The elemental analysis of the Chi sample was performed in an organic elemental analyzer (INICUBE**®,** Elementar Americas Inc.) designed for simultaneous carbon, hydrogen, nitrogen, and sulfur analysis in solid and liquid samples. This analysis allows the detection of differences in the presence of sulfur atoms between Chi-S and Chi-C, which indicates sulfation degrees of Chi-C. Furthermore, Fourier transform infrared (FTIR) spectra of Chi-S and Chi-C were obtained with an ATR/FTIR interspec 200-X spectrometer. The FTIR spectra were recorded at 4 cm^−1^ resolutions and 64 scans. Additionally, surface morphology and micro-analysis of Chi-S and Chi-C samples were observed by scanning electron microscopy (SEM model JSM-IT300LV, JEOL USA Inc.), coupled with energy dispersive X-ray analysis (EDS Aztec, Oxford Instruments); the elemental analysis was performed using a computer-controlled software (Aztec analysis software, Oxford Instruments).

### Viruses and cells

PCV2 genotype b (strain 0233) was passed three times and titrated on PK-15 cells as previously described [24]. PK-15 cell line (ATCC CCL-33, passage 10) was maintained in minimal essential medium (MEM) (Corning™ Cellgro™), supplemented with 15% fetal bovine serum (Corning Cellgro), 0.5% antibiotic/antifungal (10,000 IU/mL penicillin, 10,000 µg/mL streptomycin and 25 µg/mL amphotericin B and 1% glutamine (Corning™ Cellgro™). Aliquots of the virus were stored at − 80 °C until required.

### Cell seeding and infection of cell cultures

PK-15 cells were seeded at 2.0 × 10^4^ cells /well in 96-well microplates (Corning™ Cellgro™) and incubated for 12 h at 37 °C/5% CO_2_ until 80% confluence. Before adding Chi samples or the virus, or when quantifying the results, the monolayers were washed twice with phosphate buffered-saline (PBS, pH 7.4 at room temperature). In all the experiments the following controls were included: cell control (cells that were not infected with the virus or treated with Chi samples); virus control (cells that were infected only with the virus but not treated with Chi samples in the antiviral activity assays).

### Cytotoxicity assay

The cytotoxicity of the polymers was determined using the method described by Pourianfar et al*.* [25] with modifications. Briefly, the media of the 80% confluent PK-15 cells was aspirated and replaced with 100 µl of each polymer solution diluted in MEM/15% FBS at concentrations of 0, 5, 10, 15 and 20 mg/mL. After incubation at 37 °C/5% CO_2_ for a further 3 days, the results were quantified using CellTiter 96® AQueous One Solution MTS (Promega Corporation), with the absorbance set at 490 nm, according to the manufacturer’s instructions. Cell viability percentages were measured based on the number of living cells in polymer-treated cells relative to cell controls (defined as 100% viability). The cytotoxicity curve was then generated by plotting cell viability percentages against compound concentrations.

### Antiviral activity assay

The infectivity of PCV2 on PK-15 cell cultures treated with LMW and HMW Chi-S was evaluated through a plaque infection assay. The monolayers were infected with 10 µL of PCV2 inoculum (10^6^ TCID_50_ mL^−1^, MOI = 5), then the microplate was incubated at 37 °C/5% CO_2_ for 1 h. After the incubation time, the residual viral inoculum was removed by washing the cell monolayers with 1X PBS and immediately covered with 100 µL of MEM supplemented with 15% FBS, 0.5% antibiotic/antifungal and 1% glutamine, which contained five concentrations of Chi-S or Chi-C (0, 5, 10, 15 and 20 mg/mL). The incubation continued for 24 h at 37 °C/5% CO_2_, after which the supernatant was removed and the cell cultures were treated with D-glucosamine (300 mM) and incubated for additional 48 h. After the incubation period, the monolayers were subjected to 3 freeze–thaw cycles (− 80 °C) and the lysates of all wells were obtained and subsequently centrifuged at 2500 g to store the supernatants for quantification of viral DNA and TCID_50_ titer.

### Viral copy number measuring by quantitative real-time PCR (qPCR)

Viral DNA was extracted according to the manufacturer's instructions (Axigen, Biosciences). PCR was performed using primers that amplify a specific region of PCV2 ORF2 (PCV2qPCR-F 5′-ATGTCCACCGCCCAGGAGG-3′ Position 1523-1541 and PCV2qPCR-R 5′-CCGYTGGAGAAGGAAAAATGGCATC-3′ Position 1603-1627). The SYBR Green system (Kapa Sybr) and a real-time thermal cycler (LightCycler Nano, Roche) were used for real-time PCR, following the manufacturer's instructions. To generate the standard curve, the plasmid pGEM::PCV2 was used, in which the complete PCV2 DNA genome sequence was cloned. The plasmid DNA was extracted from the bacterial strain *E. coli* DH5α, with a commercial kit (Plasmid Midi Kit, Qiagen), according to the manufacturer's instructions. The quantification was conducted by measuring the OD_260_ using NanoDrop spectrophotometer and DNA copies by using the online NEBio calculator (https://nebiocalculator.neb.com/#!/ligation). Serial dilutions (10^1^–10^5^) were prepared in duplicate of known molar concentrations of plasmid DNA. The viral DNA copies were interpolated from the standard curve obtained. The degree of inhibition of the virus was recorded by quantify the genomic copies of PCV2 in log_10_ DNA copies/mL.

### Quantification of virus titer

The quantification of PCV2 titer was determined using the method described by Zhu et al. [26] with modifications. Total virus yield (supernatants from cell lysates) were determined by inoculating tenfold serial dilutions into confluent PK-15 cells in 96-well culture plates (Corning™ Cellgro™). After 72 h of incubation, supernatant was removed, cells were fixed with 80% cold acetone and the viral antigen was detected using immunofluorescence (IFA) with mouse anti-Cap PCV2-specific monoclonal antibody (isotype IgG2a, Jeno Biotech Inc.) and FITC-conjugated goat anti-mouse IgG (H + L) (Kirkegaard & Perry Laboratories Inc.). The 50% tissue culture infective dose (TCID_50_) was calculated according to the Reed-Muench method and expressed as TCID_50_/mL.

### Detection of PCV2 Cap protein by dot blot and Western blot

Samples of the supernatant (10 μl) were taken and the amount of PCV2 Cap antigen was determined using the dot blot and Wester blot as described by Bucarey et al. [27] with modifications. Briefly, samples were transferred onto a nitrocellulose membrane using a Biodot™ microfiltration apparatus (Bio-Rad, CA, USA) or resolved by 12% SDS-PAGE electrophoresis under reducing conditions (1% glycerol, 0.4% SDS, 0.1% mercaptoethanol, 12.5 mM Tris–HCl, pH 6.6) and electrotransferred onto a nitrocellulose membrane (BioRad) using a TransBlotTM Semi-Dry Transfer Cell (BioRad, USA). The nitrocellulose membrane was then blocked overnight in 5% skim milk at 4 °C and then incubated overnight at 4 °C with a mouse anti-Cap PCV2-specific monoclonal antibody (isotype IgG2a, Jeno Biotech Inc.) or anti-β-actin antibody (Sigma Aldrich®) diluted 1:100 in PBS/0.1% Tween20 (PBST). After washing with PBST, the membrane was incubated with horseradish peroxidase-conjugated goat anti-mouse IgG (H + L) (1:1000 dilution; Kirkegaard & Perry Laboratories Inc.) for 1 h. After further washing, the signal was detected using 4-chloro-1-naphthol/H_2_O_2_ as directed by the manufacturer (Pierce, Rockford, IL, USA). The concentration of PCV2 Cap protein was estimated by comparing the signal intensities of the Western blots with those of of β-actin as loading control or by comparing the signal intensities of the dot blots with those of known concentrations of a highly purified recombinant 6xhis-Cap fusion protein, using an image analysis program (ImageJ) [28].

### Attachment assay

The ability of Chi-S to inhibit viral attachment to PK15 cell line was evaluated as previously described [25] with modifications. The 80% confluent monolayers were pre-cooled at 4 °C for 1 h followed by (1) Cellular infection with 10 µL/well of PCV2 inoculum (10^6^ TCID50 ml^−1^, MOI = 5), supplemented with 100 µl of MEM containing LMW Chi-S at the following concentrations (0, 5, 10, 15 and 20 mg/mL), (2) infection with 10 µL/well with virus inoculum that had been mixed and pre-incubated with 100 µL/well of each compound at 4° C for 30 min. All plates were kept at 4 °C for another 3 h, after which the monolayers were washed twice with PBS to remove excess compounds and any unbound viruses. The plates were then filled with 100 μl/well MEM and incubated for 24 h at 37 °C/5% CO_2_, at which point the supernatant was removed and the cultures were treated with D-glucosamine (300 mM), after which the incubation was continued in the above-mentioned conditions until completing 48 h. After incubation, the monolayers were subjected to 3 freeze–thaw cycles (− 80 °C) and the lysates of all wells were obtained and centrifuged at 2500 g to store the supernatants and quantifying the genomic copies and titers of PCV2.

### Viral penetration assay

The viral penetration assay was conducted using the modified penetration test as previously described [25], in order to evaluate the ability of the compounds to inhibit viral entry to the cells. Confluent cells (80%) were pre-cooled and infected with 10 µL of PCV2 inoculum (10^6^ TCID_50_ ml^−1^, MOI = 5) and incubated at 4 °C for 3 h to allow viral attachment. After this step, the cells were treated at room temperature with 100 µL/well of LMW Chi-S using the same concentrations (0, 5, 10, 15 and 20 mg/mL), followed by incubation at 37 °C/5% CO_2_ for 20, 40, and 60 min (separate plates) to allow the compound to interrupt the penetration of virus into cells. Each time point was tested separately and independently. The supernatant was aspirated followed by washing the cells with alkaline PBS (pH 11) for 1 min to inactivate viruses that did not penetrate the cell, after which, acidic PBS (pH 3) was added to neutralize the pH. Then, the PBS was aspirated and neutralized by washing the cells with PBS (pH 7.4) and the cells covered with 100 µL of MEM. The plates were incubated at 37 °C with 5% CO_2_ for 24 h at 37 °C/5% CO_2_, where the supernatant is removed and the cultures are treated with D-glucosamine (300 mM), continuing with incubation in the above-mentioned conditions until completing 48 h. After incubation, the monolayers were subjected to 3 freeze–thaw cycles (− 80 °C) and the lysates of all wells obtained, centrifuged at 2500 g. Supernatants were kept for quantifying genomic copies and titers of PCV2.

### Statistical analysis

Each Chi concentration was tested in triplicate. Data were expressed as mean ± standard deviation (SD). The reduction in viral DNA copies and titers were determined by the difference between the average value of DNA copies or TCID_50_ of PCV2 obtained in the control (without polymer) and the average value of viral DNA copies and titers obtained with all 5,10, 15, and 20 mg/mL concentrations of Chi-C or Chi-S polymers. The reduction in the genomic copies and titers of PCV2 were expressed as a percentage, using Eq. (1).

1 $$\begin{aligned} & \frac{{10^{{\left( {\text{LC Concentration}} \right)}} { }}}{{10^{{\left( {\text{LV Control}} \right)}} }} \times 100 = {\text{X}} \\ & 100 - {\text{X}} = {{\% }}\;{\text{PCV}}2\;{\text{reduction}} \\ \end{aligned}$$

where LC concentration and LV control correspond to the average of log_10_ DNA copies/mL or log_10_ TCID_50_/mL obtained at each polymer concentration and the average value obtained in the control (without polymer), respectively. The results of the antiviral activity tests were previously analyzed with an ANOVA test using a multivariate regression model. Tukey's honest significant difference test (HSD) was then used to compare the antiviral activity between the type and concentration of Chi. A significance level (*p* value) set to ≤ 0.05 and ≤ 0.005 were considered to compare the group means. All the graphs, calculations, and statistical analyses were performed using GraphPad Prism software (GraphPad Software, San Diego, CA).

## Results

### Characterization of Chi-S and Chi-C samples

**Fig. 1:**
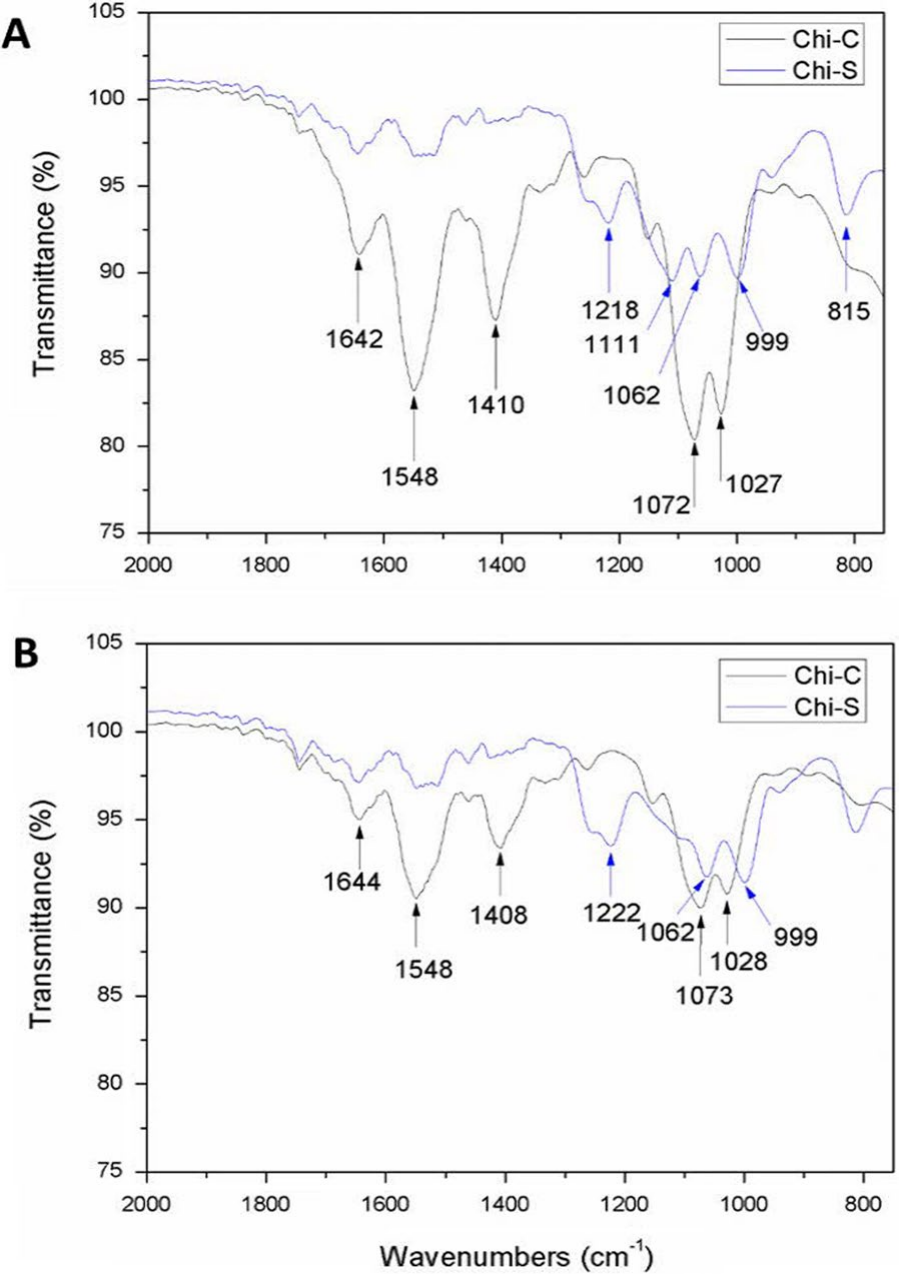
Illustrate the infrared spectra (FTIR) obtained for LMW Chi-S (blue line) versus LMW Chi-C (black line) (panel **A**) and HMW Chi-S (blue line) versus HMW Chi-C (black line) (panel **B**) using a ATR/FT-IR interspec 200-X spectrometer. Blue arrows show specific absorption bands at 815, 999 and 1218 cm^−1^ corresponding to the C–O–S and O=S=O. LMW Chi-C, low molecular weight commercial chitosan; LMW Chi-S, low molecular weight sulfated chitosan; HMW Chi-S, high molecular weight sulfated chitosan

The presence of sulfur (Tables 1 and 2) and the sulfate groups (Fig. 1) of Chi-S samples was confirmed by elemental analysis and FTIR spectroscopy, respectively. Table 1 presents the results obtained from the elemental analysis of LMW Chi-S, a theoretical elemental composition of Chi-C and reported raw material of Chi-C. However, similar results showing Chi-S have been reported by [29] (4.13% N, 21.62% C, 4.53% H and 12.91% S).

**Table 1:** Elemental analysis of Chi and Chi-S

Sample	%N	%C	%H	%S
LMW Chi-S	5.22 ± 0.15	26.62 ± 0.61	5.02 ± 0.27	8.5 ± 0.54
Chi-C	8.7*	44.7*	6.8*	0.0*
Chi^a^	7.51	40.04	7.11	0.0

The elemental analysis represents an average of three measurements

N, nitrogen; C, carbon; H, hydrogen; S, sulfur. LMW Chi-S, low molecular weight sulfated chitosan; Chi-C, commercial chitosan; Chi, chitosan

*Theoretical idealized elemental composition of Chi-C

^a^[30]

**Table 2:** EDS measurements of LMW and HMW of Chi and S-Chi samples

Sample	%N	%C	%O	%S	%Na	Total wt%
LMW Chi	8.1	50.54	41.36	–	–	100
LMW Chi-S	–*	37.76	44.28	10.82	7.14	100
HMW Chi	8.08	49.46	42.46	–	–	100
HMW Chi-S	7.56	33.43	43.63	8.77	6.6	100

C, O, N, S, and Na are the percentage amounts of carbon, oxygen, nitrogen, sulfur and sodium atoms in the Chi sample

N, nitrogen; C, carbon; H, hydrogen; S, sulfur; wt, weight; LMW Chi, low molecular weight chitosan; LMW Chi-S, low molecular weight sulfated chitosan; HMW Chi, high molecular weight chitosan; HMW Chi-S, high molecular weight sulfate

*Atom is present but was not measured

–Absence of the atom

Comparing the FTIR spectra of LMW and HMW Chi-S and Chi-C different absorption bands appears due to the sulfation reaction. The FTIR spectrum of Chi-C exhibited the characteristic bands at 1642 cm^−1^ (amide I), 1548 cm^−1^ (amide II) and 1410 cm^−1^ (amide III). Additionally, the characteristic bands are observed at 1027 and 1072 cm^−1^ corresponding to the stretching of C–O. However, for Chi-S the FTIR shows new absorption bands at 815 and 1218 cm^−1^ corresponding to the C–O–S and O=S=O groups, respectively, confirming that the sulfation reaction was successful and that these bands are characteristic of the sulfate groups in the Chi-S chains [31]. Indeed, the band around 815 cm^−1^ is attributed to the vibration of the stretching of C-O-S and the band at 999 cm^−1^ can be assigned to the sulfate group [31] (Fig. 1A, B).

**Fig. 2:**
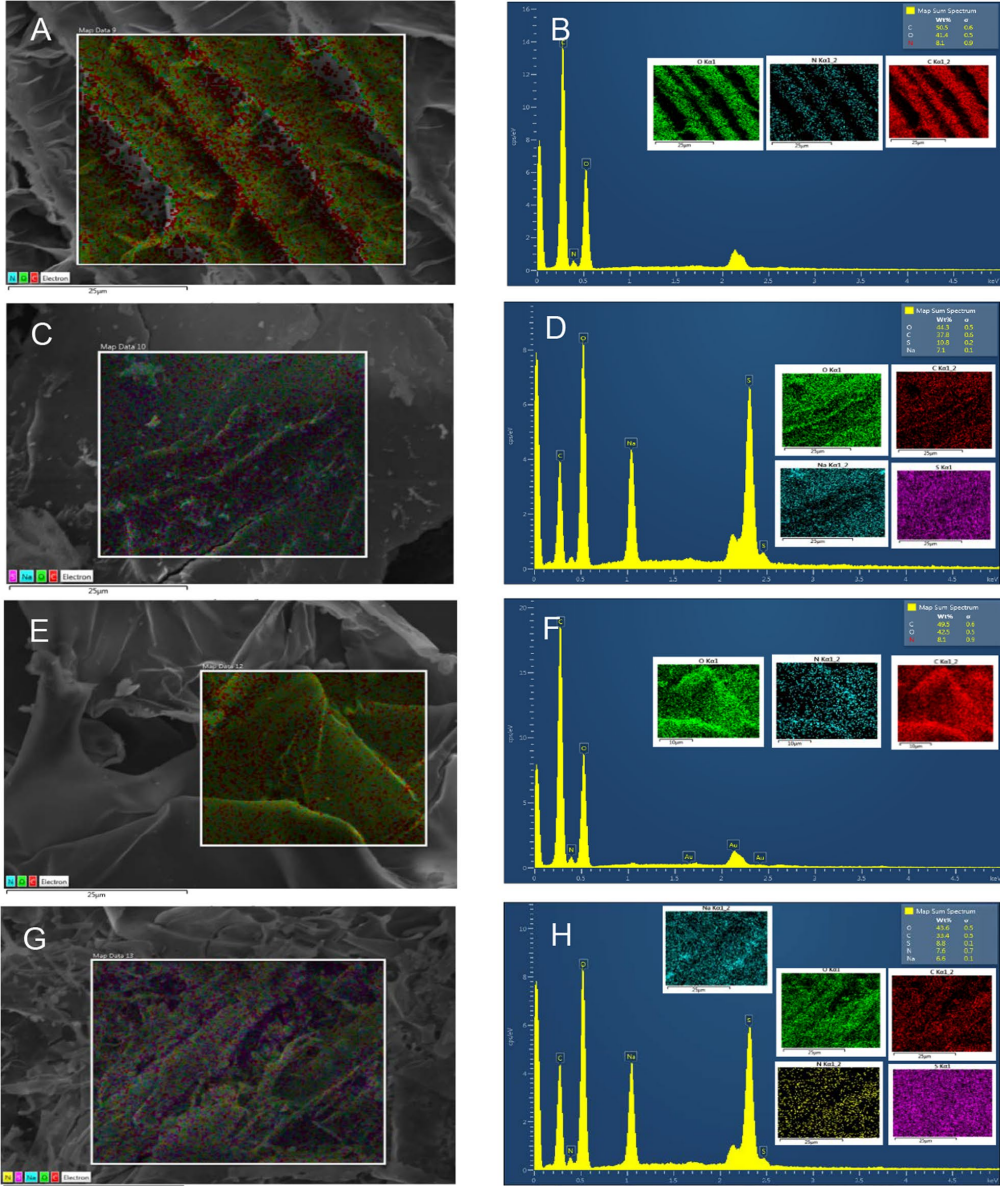
Surface morphology and micro-analysis (SEM–EDS) of Chi-C and Chi-S samples. LMW Chi-C (panel **A**, **B**); LMW Chi-S (panel **C**, **D**); HMW Chi-C (panel **E**, **F**) and HMW Chi-S (panel **G**, **H**). The colored represents elemental composition of analyzed region on the sample (green = oxigen; blue = nitrogen; red = carbon; pink = sulfur)

Additionally, surface morphology and micro-analysis of Chi was observed by SEM EDS analysis (Fig. 2). SEM images of representative Chi samples show homogenous surfaces of typical raw Chi material. Indeed, EDS measurements were performed to determine the elemental composition of LMW and HMW, in their Chi-S and Chi-C forms. We determined that in the absence of sulfate groups of LMW and HMW Chi samples, the chemical composition of Chi did not either show a sulfur atom nor differ from theoretical predictions regarding to unmodified raw Chi. Therefore, demonstrating that for both LMW and HMW Chi-C samples, the sulfur atom was absent. However, when EDS measurements were performed on functionalized LMW and HMW Chi-S in both cases the presence of sulfur atoms was consistently shown in the range of 10–8 wt%, respectively, as observed in Table 2.

### Molecular weight dependence of antiviral activity of sulfated chitosan

**Fig. 3:**
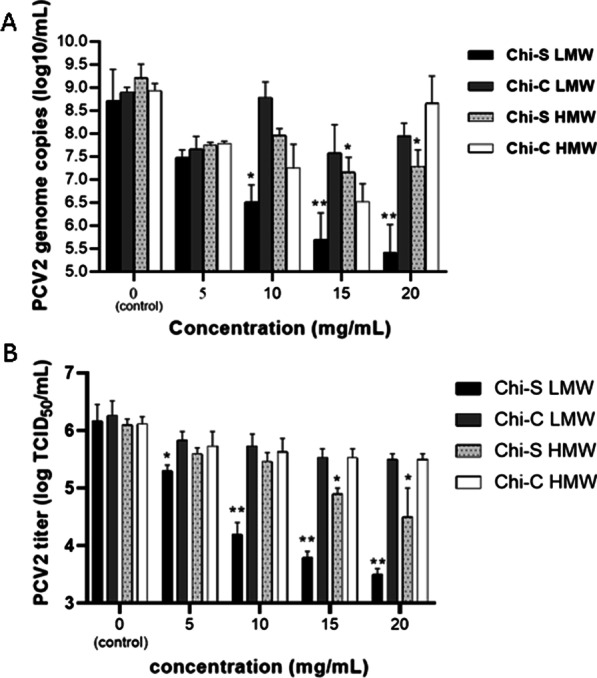
Antiviral effect of Chi-C and Chi-S samples. PK-15 cell monolayers were infected with 10 µL of PCV2 suspension (10^6^ TCID_50_ ml^−1^, MOI = 5), after one hour incubation, the cells were exposed at 0, 5, 10, 15, and 20 mg/mL of LMW Chi-C; LMW Chi-S; LMW Chi-S and HMW Chi-S. The results are presented as log_10_ viral DNA copies/mL (Panel **A**) and log TCID_50_/mL (Panel **B**). Data are expressed as the mean ± SD generated from three replicates and a significant differences set to *p ≤ 0.05 and ** *p* ≤ 0.005 were considered

When analyzing the antiviral activity of Chi, the values obtained from the viral DNA copies and titers with LMW Chi-S and Chi-C show significant differences. In the case of LMW Chi-S, all concentrations reduced the DNA copy numbers and TCID_50_ titers. Indeed, 15 and 20 mg/mL of LMW Chi-S were more significant, reducing both parameters by 2.02 (99.05%) and 2.3 log (99.50%) in the case of DNA copyes and 2.36 (99.56%) and 2,66 log (99.78%) in the case of TCID_50_ titers (see Fig. 3A, B).

**Fig. 4:**
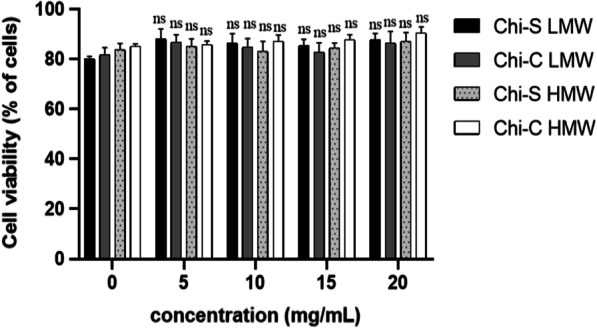
Chitosans samples have no cytotoxic effect on PK15 cells. PK15 cells were pretreated with different concentrations of Chi-S and Chi-C for 24 h at 37 ℃, and then add 10 μL of CellTiter 96® AQueous One Solution MTS (Promega Corporation) to each well for 1 h at 37 ℃ and measured absorbance at 490 nm. All data were presented as means ± SD and were analyzed using one-way ANOVA (ns = no significant)

The LMW Chi-C did not exhibit a statistically significant effect. Moreover, when comparing the HMW Chi-C, no significant differences were obtained. However, for HMW Chi-S, we observed that at concentration of 20 mg/mL, viral DNA and titers were significantly reduced by 2.02 log_10_ DNA copies/mL (99.04%) and 1,6 log_10_ TCID_50_/mL (99,60%), respectively (Fig. 3A, B). When comparing HMW and LMW Chi-S, the antiviral effects of LMW Chi-S were higher and consistantly significant with lowest viral DNA copy number and TCID_50_ titer. None of the Chi polymers showed significant cytotoxicity at concentrations 5–20 mg/mL, at which they demonstrated effective antiviral activities (Fig. 4).

**Fig. 5:**
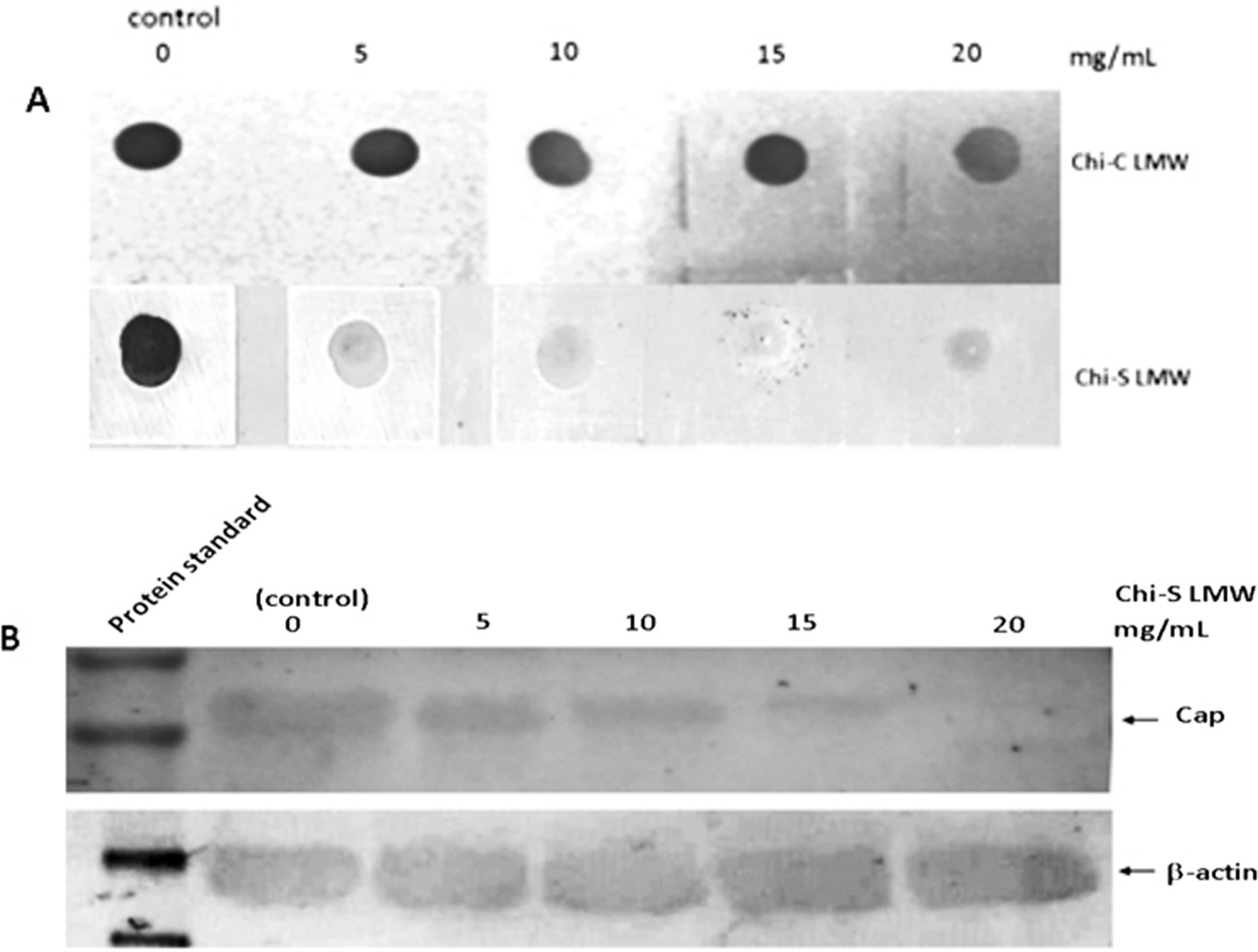
LMW chitosan sulfate inhibited PCV2 Cap protein expression in PK15 cells. Cells monolayers were infected with 10 µL of PCV2 suspension (10^6^ TCID_50_ ml^−1^, MOI = 5), and then the cells were exposed at 0, 5,10,15, and 20 mg/ml of LMW Chi-S, then PCV2 Cap protein was detected by dot blot comparing with LMW Chi-C treatment as control (panel **A**) and Western blot using β-actin as loading control (panel **B**)

In order to provide more data to determine the interaction of LMW Chi-S and viral proteins and discuss the possible role of Chi-S in viral inhibition, we used dot blot and Western blot to analyze the expression of PCV2 capsid protein (Cap) in the cells. The results showed that LMW Chi-S reduce the expression of Cap protein in a dose-depedent dependent manner, with an inhibition rate of 90 and 99% at concentrations of 15 and 20 mg/mL, respectively (Fig. 5A, B).

### LMW sulfated chitosan blocks viral cell attachment

**Fig. 6:**
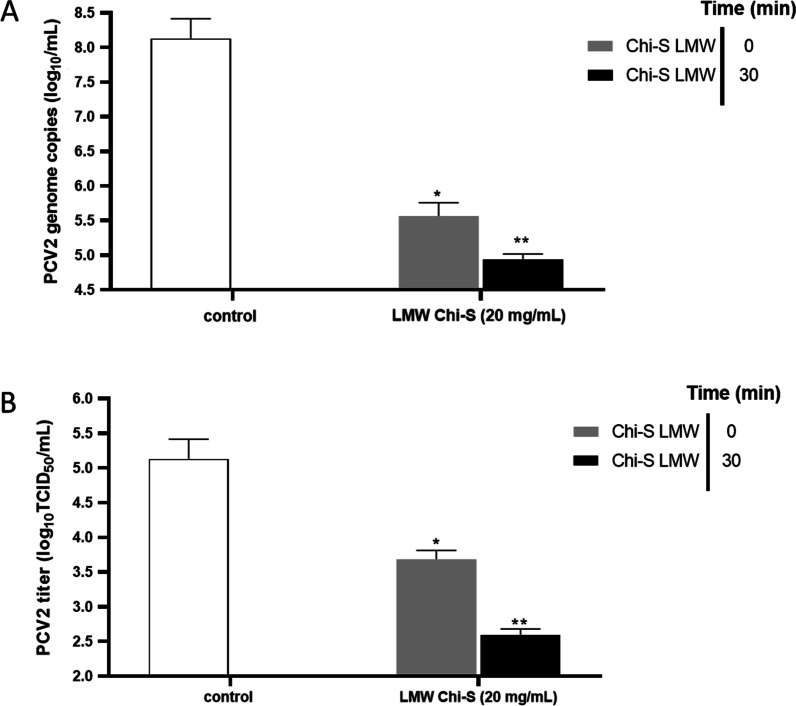
Viral attachment assay. Confluent PK15 cell monolayers were pre-cooled at 4 °C for 1 h followed by (1) Cellular infection with 10 µL/well of PCV2 suspension (10^6^ TCID_50_ ml^−1^, MOI = 5), supplemented with 100 µl of MEM containing LMW Chi-S at 20 mg/mL (2) infection with 10 µL/well with virus suspension (10^6^ TCID_50_ ml^−1^) that had been mixed and pre-incubated with 100 µL/well of the polymer at 4 °C for 30 min. White bar represents virus control. The results are presented as log_10_ viral DNA copies/mL (panel **A**) and log_10_ TCID50/ml (panel **B**) are expressed as the mean ± SD generated from three replicates and a significant differences set to *p* ≤ 0.05 and ** *p* ≤ 0.005 were considered was considered

To assess the ability of the polymers to inhibit viral binding, an attachment assay was performed. The results obtained from evaluating the ability of LMW Chi-S to inhibit viral attachment revealed that there were significant differences between LMW Chi-S (20 mg/mL) and control, with Chi-S reducing the viral copy number and titer of cell cultures to a greater extent (Fig. 6A, B). When the group “without incubation” (time 0) was compared with the group “with 30 min incubation” in the presence of LMW Chi-S, it showed more significant differences. In fact, the virus-chitosan pre-incubation reduced the viral copy number and titer by 3.19 log_10_ DNA copies /mL (99.94%) and 2.56 log_10_ TCID_50_/mL (99.77%), respectively (Fig. 6A, B).

### LMW sulfated chitosan reduces viral cell penetration

**Fig. 7:**
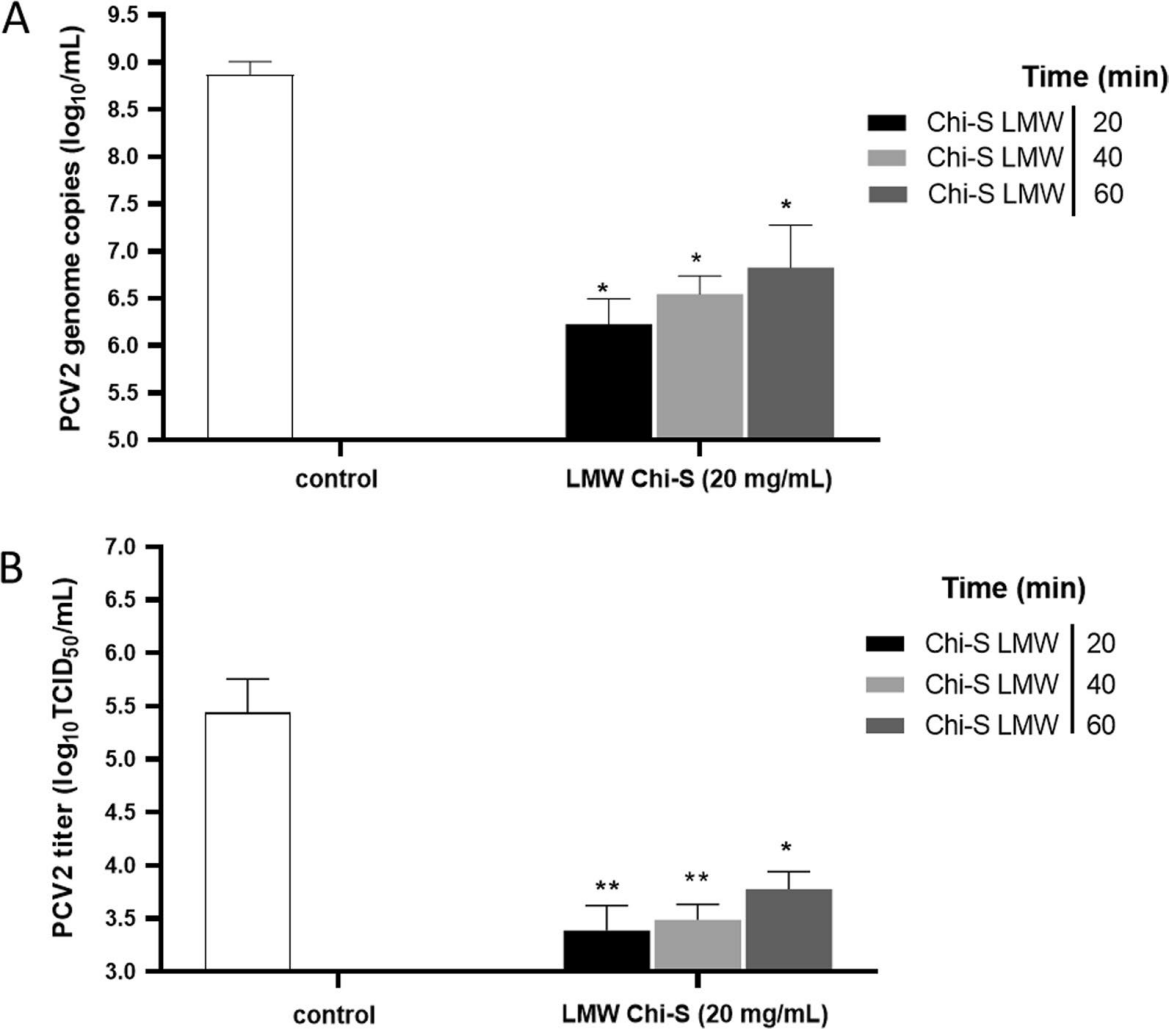
Viral penetration assay. PK-15 confluent cells were pre-cooled and infected with 10 µL of PCV2 suspension (10^6^ TCID_50_ ml^−1^, MOI = 5) and incubated at 4 °C for 3 h to allow viral attachment. After this step, the cells were treated at room temperature with 100 µL/well of each polymer using the same four concentrations (0.5,1.0,1.5, and 2.0%), followed by incubation at 37 °C/5% CO_2_ for 20, 40, and 60 min (separate plates) to allow the compound to interrupt penetration of virus into cells. Each time point was tested separately and independently. White bar represents virus control. The results are presented as log_10_ viral DNA copies/mL (panel **A**) and log_10_ TCID50/ml (panel **B**) are expressed as the mean ± SD generated from three replicates and a significant differences set to *p* ≤ 0.05 and ** *p* ≤ 0.005 were considered was considered

The penetration test was used to evaluate the ability of LMW Chi-S to inhibit viral entry. The results reveal that low molecular weight sulfated polymer could significantly reduce the viral copy number and titer. LMW Chi-S applied for 20 min significantly reduced the viral copy number and titer when it was used at 20 mg/mL, presenting reduction of 2.64 log_10_ copies/ml (99.77%) and 2.05 log_10_ TCID_50_/mL (99.11%). Application of LMW Chi-S for 40 min also produced significant effect, reducing the viral DNA by 2.33 log_10_ copies/mL (99.53%) and titers by 1.95 TCID_50_/mL (98.87%). Finally, when Chi-S for was applied for 60 min, the effect was less significant and reduced the viral copy number by 2.05 copies/mL log_10_ (99.11%) and titer by 1.66 TCID_50_/mL (97.81%). In general, the reduction of viral DNA copies and titers, in the penetration assays, did not depend on the time of Chi-S treatment, however this reduction was less effective with 60 min treatment, compared with 20 and 40 min of treatement (Fig. 7).

## Discussion

According to many studies, sulfated polysaccharides are a promising source of antiviral molecules. In effect, in this study Chi-S was found to inhibit the in vitro viral infection of PCV2, indicating that the presence of sulfate groups improves the antiviral activity of Chi. Other factors influencing the antiviral activity would be: the presence of anionic groups, the degree of sulfation and molecular weight, affecting the virus penetration by altering intracellular signals and delaying the replicative cycle of the virus [32]. Other studies indicated that hypersulphated polysaccharides interfere with electrostatic interactions between the positively charged region of the viral envelope glycoproteins and the negative charges of the heparan sulfate surface receptor chains [33].

It was observed in this study that the LMW Chi-S produces the lowest viral copy number and titer among all polymers tested (Fig. 3A, B), with no cytotoxic effects (Fig. 4).

LMW Chi-S also reduces the expression of capsid protein, Cap, in a dose-dependent manner (Fig. 5A, B). Cap is the only PCV2 structural protein, which is involved in diverse and essential biological events during virus infection, such as virion attachment [34]. A possible explanation of this effect is that the depolymerization of the polysaccharide during the sulfation process allows it a better interaction with the capsid structure, generating an antiviral effects and therefore inhibiting the establishing further interactions with the cell. However, at present there is no clear consensus about the effect of molecular weight on the antimicrobial properties of Chi, indeed, some conclusions are contradictory [35]. According to Jaber et al. [36], the antiviral activity of Chi increases as its molecular weight decreases and as its degree of acetylation increases. Thus, data from other studies indicate that HMW Chi possesses higher antiviral properties [36, 37].

It is possible that inhibition of the virus using Chi-S occurs during the initial stages of infection, supporting Chi-S as a good antiviral agent. Therefore, if the virus-receptor interaction is hindered, processes such as attachment, signaling, internalization, endocytosis, and replication would be affected [32].

The results obtained are similar to those reported for other polysaccharides, where it is deduced that the most acceptable mechanism of action is impeding the viral attachment of PCV2 to sulfated polysaccharide receptors. Thus, when heparan sulfate and Chi-S are chemically compared, structural analogy is obseved and consequently they can mimic their biological functions [7, 8].

It was also observed that pre-incubating the virus with Chi-S produced a greater reduction in viral copy number and titer. In Addition, although less significant, applying the Chi-S at the time of infection also produced reduction in viral copy number and titer (Fig. 6A, B). Therefore, it could be established that there is a blockage of viral adsorption through direct interference with the virus particle. It has been reported that 3,6-sulfated Chi directly inhibits human papillomavirus (HPV) by binding to viral capsid proteins and, therefore, blocking viral adsorption [38]. Other sulfated Chi-oligosaccharide derivatives block the interaction between HIV-1gp120 and CD4 + cell surface receptors, thereby inhibiting virus-cell fusion and subsequent virus entry. This induces a masking effect that eventually inhibits the attachment and subsequent penetration of the virus into host cells [39]. Recently, it was demonstrated that sulfated polysaccharides possess a strong antiviral effect due to their direct interaction with SARS-CoV spike protein [32]. Our results indicate that LMW Chi-S could interact directly with viral capsid of PCV2 to inactivate it, and thus allow antiviral responses. In support of our evidence, it was reported that low-molecular-weight sulfated chitosan, naturally obtained from the squid *Sepia pharaonis*, presents antiviral activity against Newcastle disease virus by binding to the surface glycoprotein [40]. Therefore, its antiviral activity would be mediated by a competitive inhibition of the attachment of the viral particle to avian erythrocytes, thus functioning as a potential antiviral agent. Our results suggest that the Chi-S chemically obtained from industrial waste has great potential as an anti-PCV2 product that can be further validated in vivo.

According to our results, significant reductions of viral copy numbers and titers were observed in the early stages of infection. Therefore, Chi-S interferes with the penetration of the virus, since a reduction in viral DNA and titer were also observed when viral adsorption in cell culture was allowed (Fig. 7A, B). These reductions were important in the first 40 min. However, at 60 min a reduction in viral DNA was still observed, but not to such an efficient extent, as it was for early post-infection stages (Fig. 7A, B).

Recent research indicates that sulfated polysaccharides interfere with the internalization of coronavirus by interacting with its membrane proteins, specifically, by binding with carbohydrate groups linked to the polypeptide chains of the virus to inhibit their penetration [41]. Additionally, sulfated polysaccharides bind to the allosteric site of the viral capsid, preventing the virus from initiating its cycle within the host cell. Indeed, Lee et al., 2004, investigated sulfated polysaccharides from green algae and synthetic sulfated xylan exhibit potent antiviral activities against HSV-1 (herpes simplex virus) [42]. The authors showed that some sulfated polysaccharides not only inhibited the early stages of viral cycle, such as viral attachment and penetration into host cells, but also interfered with the late stages of replication.

We have previously described the ability of generic Chi-C (LMW) to bind with PCV2 virus-like particles and that this conjugate would be useful and efficient for developing mucosal vaccines against PCV2 virus, since Chi, is an excellent adjuvant of the mucosa-associated immune system [27].

In this study, we have shown that LMW Chi-S exhibits significant anti PCV2 activity. The mechanism of action would be through inhibition of viral adsorption, acting as a mimetic of the heparan sulfate receptor, binding to the viral capsid proteins and therefore blocking the attachment of the virus to the host cell, which reflects that Chi-S can be considered a “clone” coreceptor for virus binding, which would compete in the attachment with heparan sulfate or chondroitin sulfate, the virus natural receptors on the cell surface. It has been well demonstrated that PCV2 uses these GAGs for attachment to porcine monocytic 3D4/31 and PK-15 cells [34].

## Conclusions

Our work suggests that LMW Chi-S would affect early stages of the viral cycle, such as attachment or penetration into the host cells. Although sulfated polysaccharides have been extensively studied for a long time in search of evidence of their antiviral properties, no previous research has been conducted regarding the antiviral activity of Chi-S on PCV2 infection. Therefore, we highlighted the potential of this biopolymer as a specific therapeutic compound. However, more research focus on molecular interaction between PCV2 virion and LMW Chi-S should be addressed in the future to facility a more comprehensive mechanism of the antiviral activity and potential of LMW chitosan sulfate. This warrants further studies to fully investigate the specific mechanisms that LMW Chi-S plays in preventing PCV2 infection in vivo.

## Data Availability

The datasets used and/or analysed during the current study are available from the corresponding author on reasonable request.

## Abbreviations

Chi: Chitosan
Chi-C: Commercial chitosan
LMW Chi: Low molecular weight chitosan
HMW Chi: High molecular weight chitosan
LMW Chi-S: Low molecular weight sulfated chitosan
HMW Chi-S: High molecular weight sulfated chitosan
SEM–EDS: Scanning electron microscopy-energy dispersive X-ray spectroscopy
FT-IR: Fourier transform infrared spectra
DS: Degree of sulfation
MEM: Minimal essential medium
